# Neurofeedback strategies in binge-eating disorder as predictors of EEG-neurofeedback regulation success

**DOI:** 10.3389/fnhum.2023.1234085

**Published:** 2023-10-30

**Authors:** Jytte Wimmer, Sarah Alica Rösch, Ricarda Schmidt, Anja Hilbert

**Affiliations:** ^1^Integrated Research and Treatment Center Adiposity Diseases, Behavioral Medicine Research Unit, Leipzig University Medical Center, Leipzig, Germany; ^2^International Max Planck Research School NeuroCom, Leipzig, Germany

**Keywords:** EEG, mental strategy, brain activity, obesity, binge-eating disorder, neurofeedback, cognitive control, beta treatment

## Abstract

**Introduction:**

Treatment options such as neurofeedback (NF) that directly target the link between aberrant brain activity patterns and dysfunctional eating behaviors in binge-eating disorder (BED) are emerging. However, virtually nothing is known about mental strategies used to modulate food-specific brain activity and the associated brain-based or subjective success of specific strategies. This study firstly investigated the use of mental strategies in response to individually appetitive food cues in adults with BED and overweight or obesity based on a randomized-controlled trial providing electroencephalography (EEG)- or real-time functional near-infrared spectroscopy (rtfNIRS)-NF to BED.

**Methods:**

Strategy reports written by participants were classified with qualitative content analysis. Additionally, the mental strategies employed by the *N* = 23 patients who received EEG-NF targeting the reduction of fronto-central high beta activity were analyzed quantitatively through their link with subjective and EEG-NF regulation success.

**Results:**

The following eight categories, ordered by frequency in descending order, were found: “Behavior,” “Imagination,” “Emotion,” “Distraction,” “Thought,” “Concentration,” “Self-Talk” and “No Strategy.” Linear mixed models revealed “Imagination,” “Behavior,” and “Thought” strategies as positive predictors of EEG-NF regulation success (defined as high beta activity during regulation beneath the baseline), and “Concentration” as a negative predictor of subjective (i.e., self-reported) NF regulation success.

**Discussion:**

In conclusion, our study offers a classification system that may be used in future studies assessing strategy use for regulating food-related responses in patients with BED and associated overweight/obesity, providing valuable information on potential benefits of specific strategies and transferability to situations outside the NF treatment.

## 1. Introduction

Binge-eating disorder (BED; American Psychiatric Association, 2013) is the most prevalent eating disorder in adults, with a mean lifetime prevalence of up to 2.8% (Galmiche et al., 2019). BED is defined by recurrent episodes of binge eating involving eating unambiguously large amounts of food in a discrete period of time, accompanied by a sense of loss of control over eating. Regular compensatory behaviors to prevent weight gain are absent contrary to bulimia nervosa. BED co-occurs with mental disorders, including major depressive disorder, and somatic disorders such as Type 2 diabetes mellitus and essential hypertension (Udo and Grilo, 2019). Cognitive-behavioral therapy is considered as first-line treatment for BED in evidence-based international clinical guidelines (Hilbert et al., 2017). Meta-analyzes showed large-sized reductions in binge-eating episodes when compared to inactive control conditions (Hilbert et al., 2019). However, only 46–52% of patients remained abstinent from binge eating in the long term (Hilbert et al., 2020), indicating the need for further treatment optimization.

Recent evidence suggested that aberrant brain activity patterns are implicated in the development and maintenance of BED (Aviram-Friedman et al., 2018; Donnelly et al., 2018). Specifically, a systematic review on studies using electroencephalography (EEG) revealed increased fronto-central beta activity in individuals with BED and overweight or obesity in resting state and during food cue presentation relative to healthy, normal-weight controls (Blume et al., 2019). These consistent findings initiated the development of brain-directed treatment options which directly target these neural deviations in BED (Imperatori et al., 2018; Blume et al., 2022). EEG-neurofeedback (NF) is such a brain-directed treatment approach that seeks to enable individuals to gain voluntary control over their brain functions by providing them real-time feedback on their brain activity (Yucha and Montgomery, 2008), for example via visual stimuli. Building on operant learning, participants are reinforced if they regulate the signal in the desired direction (Sherlin et al., 2011). EEG-NF has shown potential in treating adults with BED (Blume et al., 2022), overweight and obesity (Fattahi et al., 2017; Leong et al., 2018), and subthreshold eating disturbances (Schmidt and Martin, 2015, 2016, 2020). Specifically, reductions in binge-eating episodes and eating disorder psychopathology were found at post-treatment and at 6-month follow-up of 12 sessions of food-specific EEG-NF, targeting fronto-central high beta activity, or functional near-infrared spectroscopy-based NF, targeting prefrontal cortex upregulation, in a recent randomized-controlled trial for patients with BED (Hilbert et al., 2023). Another randomized-controlled pilot study consistently demonstrated decreases of binge-eating episodes after 10 sessions of food-specific EEG-NF, addressing the reduction of fronto-central high beta (23–28 Hz) and theta activity, and at 3-month follow-up in patients with BED (Blume et al., 2022). These symptom improvements were accompanied by significant physiological reductions of high beta activity during resting state and food-cue presentation at posttreatment compared to pretreatment.

Despite these promising findings, the underlying mechanisms of EEG-NF are poorly understood. There is an ongoing debate about the factors which affect NF outcomes, including participants’ use of mental strategies (Thibault et al., 2015; Muñoz-Moldes and Cleeremans, 2020). Among the few available studies on the effects of participants’ individual strategy use on regulation success, Hasslinger et al. (2020) examined the nature and role of mental strategies in response to slow cortical potentials NF in *n* = 30 children and adolescents (9–17 years) with attention-deficit/hyperactivity disorder (ADHD) over 25 sessions. Based on semi-structured interviews conducted after each fifth session, the children’s mental strategies were grouped into four categories – cognitive, emotional, physiological, and unspecified regulation – using an inductive-deductive qualitative approach. Only patients using strategies from the cognitive (such as focusing on the task) or emotional regulation category (reaching a kind of meditative state), showed success in targeted up- and downregulation of the NF signal (i.e., physiological changes of the EEG activity) and subjective (i.e., self-reported) improvement of behavioral symptoms, while no effects were found for other strategies (Hasslinger et al., 2020).

Among healthy adults (*n* = 20), those who did not use any mental strategy after the first and last of 10-session sensorimotor rhythm NF examined written strategy reports (Kober et al., 2013) showed better learning slopes (i.e., greater correlation between session number and targeted sensorimotor rhythm upregulation) than those applying visual, auditory, cheering, relaxation, or concentration strategies based on self-report (Kober et al., 2013). A further study delivering 8 gamma enhancement EEG-NF sessions to *n* = 8 healthy women showed that concentration on the task was subjectively perceived as the most successful strategy based on written strategy reports (Khodakarami and Firoozabadi, 2020), while associations between strategy use and EEG-NF regulation success were not examined. A single session of EEG-NF on short-term memory training via alpha band upregulation in *n* = 32 healthy students demonstrated that participants who reported to use positive strategies (e.g., entertainment, love, family) showed stronger increases in alpha band activity compared to those using neutral (e.g., calculation, work, number) or negative (i.e., quarrel, anger, accident) strategies (Nan et al., 2012). Likewise, strategies applied during a single sensorimotor rhythm upregulation NF session differed between *n* = 62 individuals with a positive versus negative NF learning slope, suggesting a link between strategies and NF regulation (Autenrieth et al., 2020).

It is crucial that none of these studies investigated the use of mental strategies to downregulate high beta activity, employed food stimuli, or was concerned with treating BED. Thus, it remains to date unclear which strategies are applied by patients with BED over the course of multiple sessions of food-specific NF, and whether strategy use influences brain-based and subjective regulation success. In this context, the present study sought to explore the use of strategies in adults with BED during 12 sessions of NF in which individually salient food pictures were presented (for detail, see Hilbert et al., 2023). Based on a systematic qualitative approach, session-wise reported mental strategies were categorized, hypothesizing to identify cognitive, emotional, physiological, and unspecified strategies, in line with previous studies (Kober et al., 2013; Hasslinger et al., 2020). We aimed at determining the use of mental strategies and its relationship with objective (i.e., high beta activity during regulation beneath the baseline) and subjective (i.e., self-reported) regulation success in the EEG-NF arm, which was considered separately from the real-time functional near-infrared spectroscopy (rtfNIRS) group due to the pertinent differences in the targeted processes and because the parent study (Hilbert et al., 2023) revealed differential responses in the EEG-NF versus rtfNIRS-NF group. We expected that NF strategies would differ in modulating objective EEG-based and subjective regulation success (Autenrieth et al., 2020; Hasslinger et al., 2020).

## 2. Methods

This study was part of the single-center, assessor-blinded, randomized-controlled, three-armed feasibility study of rtfNIRS-NF for BED (NIRSBED; Hilbert et al., 2023; DRKS00014752, www.drks.de). The total sample of *N* = 78 individuals was randomized to 12 60-min sessions of rtfNIRS-NF, delayed rtfNIRS-NF, or high-beta EEG-NF, delivered over 8 weeks. In agreement with the Declaration of Helsinki, this project was approved by the local Ethics Committee of the University of Leipzig (474-ek). Details were reported elsewhere (Hilbert et al., 2023).

### 2.1. Participants and procedure

In this study, strategy categories were derived across randomization arms based on all available strategy reports from *n* = 63 patients who had at least completed 2 NF sessions, considering the inclusion criteria of the present study (see Supplementary material). Accordingly, the sample of the present study was predominantly female (*n* = 49 women, 77.78%) and had overweight (25 kg/m^2^ ≤ body mass index [BMI] ≤ 30 kg/m^2^) or obesity (BMI > 30 kg/m^2^) and a diagnosis of full-syndrome BED. The association between these strategy reports and brain-based regulation success was determined in *n* = 23 patients randomized to EEG-NF.

### 2.2. Treatment

During each of the 12 sessions of EEG- or rtfNIRS-NF, patients were asked to voluntarily modulate brain signals towards individually appetitive food stimuli (see Supplementary material for details). Patients were informed that they were given feedback on their brain activity during EEG-NF treatment, with their high beta activity depicted as a bar they were supposed to decrease upon instructions. This was supposed to help them develop strategies to gain control over their eating behavior. Each EEG-NF session was comprised of (a) a 180 s baseline, during which patients were instructed to keep their eyes open and look at the fixation cross in the middle of the screen, (b) twelve 60 s NF regulation trials, during which patients were instructed to decrease bars displayed on the screen below a yellow line (their baseline) and were given real-time feedback via the bar size, (c) twelve 25 s food presentation trials, during which patients were shown food pictures in order to imagine the food as vividly as possible, and (d) six 60 s transfer trials, during which patients were asked to regulate brain activity but did not receive real-time feedback. Without any strategies suggested to them, patients were encouraged to try and find strategies that worked for them, while being allowed to use as many strategies as they liked during regulation trials.

### 2.3. Assessment

Mental strategies were extracted from patients’ written reports in a free-text format obtained after each NF session across randomization arms. As patients were allowed to use as many strategies as they liked to regulate brain activity, most patients reported the use of numerous mental strategies throughout each NF session. Consequently, the number of mental strategies varied between sessions and participants. To determine brain-based regulation success for the EEG-arm the NF therapist (a master’s or PhD level clinical psychologist) manually noted the mean amplitude of the baseline and of each NF regulation and transfer trial for high beta activity in μV during each session. Consequently, brain-based regulation success in the present study was assessed through two tasks, first through the success when getting immediate feedback in the NF regulation task and second through the success when getting delayed feedback in the NF transfer task. Supplementary Figure S2 presents an overview of brain-based success over the time course of the 12 NF sessions. Subjective regulation success was determined via patients’ rating of their perceived success in voluntarily regulating brain activity assessed after each session on a 7-point Likert scale from 1 = *not at all* to 7 = *very strong*, with higher scores indicating greater subjective success. Supplementary Figure S3 presents an overview of subjective success over the time course of the 12 NF sessions.

### 2.4. Data analytic strategy

#### 2.4.1. Qualitative analysis of strategy reports

Qualitative content analysis as an established approach of qualitative analyzes (Erlingsson and Brysiewicz, 2013) was used to classify the self-reported strategies by patients of all randomization arms (see Supplementary material for a step-by-step example of the categorization process). Overarching categories sufficiently specific to capture the essence of each report and applicable to many reports at the same time were determined. A mixed approach was employed, firstly inductively deriving categories and subcategories from a subsample of *n* = 5 randomly selected patients reporting strategy use for a total of *n* = 60 sessions. The inductive part started with an in-depth reading of the strategy reports (Elo and Kyngäs, 2008) to extract possible similarities between reports. Each fully descriptive strategy sentence was narrowed down to a condensed content unit reflecting the core meaning of the sentence, consisting of a code with no more than two words. Categories then resulted from merging similar codes, with subcategories providing more detailed information on the specific content of broader categories (Erlingsson and Brysiewicz, 2017). The derived categories were discussed between JW and RS and revised as needed to best describe all available reports. If no formerly established category fit, new categories were formed. Categories were subsequently presented to the research team, consisting of three clinical psychologists (two at master’s, one at PhD level) under supervision of AH, and final adjustments were made via consensus discussion. Once a consensus was found, this category scheme was applied to the total sample of strategy reports (Catanzaro, 1988). Based on the coders’ initial assignment to subcategories (see Results), strategies were assigned to overarching categories in a dichotomous format (category used in the respective session/category not used in the respective session), irrespective of the number of subcategories used. As patients commonly reported to use multiple strategies per session, multiple categories were often assigned per session per patient. Quantitatively, we counted the occurrence of a strategy (defined as the use of an overarching category) relative to the total number of strategy reports (i.e., the sum of all sessions of all patients in each group) to account for imbalances in the number of sessions per patient (see Table 1). To determine interrater consistency, a second rater (SR) who was naïve regarding category assignment of the first rater (JW) independently rated a subset of 227/695 (32.77%) of the strategy reports of the first rater. The consistency of raters’ responses was established by Cohen’s Kappa coefficient (Cohen, 1960) for each category, comparing the respective columns of the two matrices. Values ≥0.60 correspond to substantial, values ≥0.80 to almost perfect agreement (Landis and Koch, 1977).

**Table 1:** Examples, inter-rater reliability, and frequency of use for each category and subcategory in the EEG-NF arm.

Category	Example	Cohen’s kappa coefficient	*n*	% used^a^
*Behavior*		1.00	94	40.52%
Relaxation	I tried to relax		62	26.72%
Breathing	Paid attention to breathing steadily		45	19.40%
Physical activity	Pressed tongue against upper jaw		5	2.16%
*Concentration*		0.69	30	12.93%
Concentration on task	Directing all [mental] energy toward the task		11	4.74%
Concentration on self/body	Feeling [the] feet firmly on the ground		7	3.02%
Concentration on surroundings	Focus on the text on the monitor		14	6.03%
*Distraction*		62	54	12.93%
Distraction everyday life	To-do lists in my head		21	9.05%
Distraction music/movies	Train ride with specific people that always get in Played/sang a song in my head, played a movie trailer in my head		32	13.79%
Distraction words/number*s*	Counted forwards and backwards		8	3.45%
*Emotion*		1.00	75	1.72%
Inducing positive emotions	Thoughts of nice situations with grandchild		71	30.60%
Inducing negative emotions	Negative thoughts, rejection		4	1.72%
*Imagination*		0.72	77	33.19%
Imagination of changed visual perception	Looking for people in the meals		2	0.86%
Imagination of movement	Sense of exercising/jogging		2	0.86%
Imagination of food defense	Pushing the pictures away in my thoughts		0	0.00%
Imagination of food neutral	Imagined how the food is made		16	6.90%
Imagination of food positive	No prohibitions, enjoying small portions		15	6.47%
Imagination of food negative	Concerning the sweets, I imagined all that sugar [in the depicted food] and pretended it was poison		17	7.33%
Imagination for positive self-motivation	Imagined the new, healthy life I would have after weight reduction		32	13.79%
Imagination for negative self-motivation	I saw myself as a very fat man		3	1.29%
*Self-Talk*		0.27	17	7.33%
Verbal avoidance of overeating	When [shown] unhealthy food, I thought “Go away!”		7	3.02%
Verbal directed at self	Sentences like “I am in control!,” “I decide, not the food”		11	4.74%
*Thought*		0.86	48	20.69%
Thoughts of nothing specific	Tried to empty head		42	18.10%
Thoughts of food avoidance	Seeing the food without thinking about it		7	3.02%
*No strategy*^b^	Tried several things Hard to explain		1	0.43%

EEG, electroencephalography; NF, neurofeedback; rtfNIRS, real-time functional near-infrared spectroscopy; *N* = 232. Percentage of subcategories refer to the number of reports of subcategory use relative to the total of *N* = 232 reports. Number of reports of subcategory may not sum up to the number of overarching categories because overarching categories were only assigned in a dichotomous format (yes/no).

a Because the category scheme was derived across arms (rtfNIRS-NF, EEG-NF, and delayed rtfNIRS-NF), frequency of strategy use is more equally distributed upon joint consideration of arms as opposed to the separate consideration of strategy use in the EEG-NF arm only in this table.

b The low number of participants using “No Strategy” potentially undermined the validity of inter-rater reliability analyzes.

#### 2.4.2. Quantitative data analysis

All analysis scripts are available at https://osf.io/t3pba/ and reporting was conducted in accordance with recently published best practice guidelines (Meteyard and Davies, 2020; see Supplementary Table S3). All analyzes were performed using R 3.6.0 (R Core Team, 2020), using the packages lme4 (Bates et al., 2015) and lmerTest (Kuznetsova et al., 2017). All effects were reported as significant at a two-tailed *p* < 0.05. A mixed-effects approach was applied to analyze the relationship between strategy use (predictor variable) and EEG-based regulation success (defined as the mean difference across trials in high beta amplitude between the baseline and the NF regulation or transfer task) and subjective regulation success (Zuberer et al., 2018). To this end, each strategy was dummy coded as 1 = *strategy was applied in the respective session* and 0 = *strategy was not applied in the respective session*. The number of applied strategies per session and participant was not controlled for in the analysis. Maximal models, i.e., models that contained all possible random effects, were first computed (Barr et al., 2013). Random intercepts were only retained if model comparisons indicated the superiority of a model that contained the respective random intercept (as compared with ANOVA). The significance of the fixed effects was tested via *t-*tests using Satterthwaite degrees of freedom, which were chosen due to their robustness and their favorable error-control properties (Luke, 2017; Meteyard and Davies, 2020). Due to their specific variance partitioning, mixed models lack an agreed way for standardized effect sizes upon involvement of multiple random factors (Singmann and Kellen, 2019). Thus, in line with recommendations, unstandardized slope estimates were presented as effect size estimates without guidelines which lack generalizability in original psychological research (Pek and Flora, 2018).

## 3. Results

### 3.1. Descriptives

The sample (*N* = 63) was mostly female (*n* = 49, 77.78%), had a mean age of 47.43 years (*SD* = 13.27), and predominant obesity (*n* = 55, 87.30%). Descriptives were similar in the EEG-NF group (see Table 2).

**Table 2:** Descriptive characteristics of study patients.

Study characteristics	Total (*n* = 63)	EEG-NF (*n* = 23)
*Sociodemographics*		
Age, *M* (*SD*)	47.43 (13.27)	47.52 (14.44)
Female sex, % (*n*)	77% (49)	78% (18)
Education, % (*n*)		
≥ 12 years	51% (32)	52% (12)
*Clinical characteristics at baseline*		
BMI (kg/m^2^), *M* (*SD*)	36.65 (4.96)	36.55 (5.20)
Weight status, % (*n*)		
Overweight (BMI 25 – < 30 kg/m^2^)	13% (8)	13% (3)
Obesity (BMI ≥ 30 kg/m^2^)	87% (55)	87% (20)
*Eating disorder diagnosis (DSM-5)*		
BED, % (*n*)	81% (51)	83% (19)
BED of low frequency and/or limited duration, % (*n*)	19% (12)	17% (4)
*Eating disorder symptoms*		
EDE binge-eating frequency past 28 days, *M* (*SD*)	3.21 (2.90)	2.87 (1.98)
Eating disorder psychopathology (EDE-Q global), *M* (*SD*)	2.74 (0.98)	2.96 (1.05)
*Comorbidities*		
Depressive symptoms (PHQ-D), *M* (*SD*)	8.82 (4.11)	9.09 (4.96)
Anxiety symptoms (GAD-7 sum score), *M* (*SD*)	6.16 (4.31)	7.09 (4.87)
*Treatment completion*		
Attended 12 sessions, % (*n*)	81% (51)	83% (16)

BMI, Body Mass Index; DSM-5, Diagnostic and Statistical Manual of Mental Disorders, Fifth Edition (American Psychiatric Association, 2013); EDE, Eating Disorder Examination; EDE-Q, Eating Disorder Examination-Questionnaire (0–6* less favorable scores are asterisked); EEG-NF, electroencephalography. GAD-7, Generalized Anxiety Disorder 7-Item Scale (0–21*); PHQ–D, Patient Health Questionnaire–Depression Scale (0–27*).

### 3.2. Categories for strategies across NF arms

Qualitative content analysis resulted in eight categories derived from patients’ strategy reports in all NF arms: “Concentration,” “Imagination,” “Self-Talk,” “Distraction,” “Thought,” “Emotion,” “No Strategy,” and “Behavior” strategies (see Supplementary Tables S1 for a description of categories including subcategories). All categories except for the category “No Strategy” included multiple subcategories, summing up to a total of 13 subcategories. Across main categories, seven of these 13 subcategories incorporated the food pictures shown to patients during NF or were mainly focused on food (e.g., “imagined how the food is made,” or “concerning the sweets, I imagined all that sugar [in the depicted food] and pretended it was poison”). Inter-rater reliability for category assignment was substantial to almost perfect, with kappa coefficients ranging from 0.67 to 0.99 in the overarching categories (see Supplementary Table S1). Low kappa estimates of the category “No Strategy” were explained by the rare use of this category, which was only used in the first session, thus preventing adequate consistency estimation.

All strategy categories except for “No Strategy” and “Self-Talk” were used by more than 10.00% of patients (see Supplementary Table S1). Ordered by their frequency of occurrence across EEG-NF, rtfNIRS-NF and delayed rtfNIRS-NF arms, the categories were ranked as follows (in descending order, Supplementary Table S2): “Imagination,” “Behavior,” “Self-Talk,” “Concentration,” “Emotion,” “Thought,” “Distraction” and “No Strategy.” For the EEG-NF arm, the frequency of occurrence in descending order was as follows: “Behavior,” “Imagination,” “Emotion,” “Distraction,” “Thought,” “Concentration,” “Self-Talk” and “No Strategy.” To illustrate the two most frequently used categories, “Behavior” included strategies like relaxation or breathing, and “Imagination” included conjuring up healthier alternatives (subcategory “Imagination positive”) or ideas of the food gone moldy (subcategory “Imagination negative”). Across arms, patients applied strategies from an average of 1.60 (*SD* = 0.78) different categories; for the EEG-NF arm, patients applied strategies from an average of 1.71 (*SD* = 0.83) different categories. Strategy use varied considerably between patients and sessions (see Hilbert et al., 2023 for session-wise strategy use).

### 3.3. Effects of strategy use on brain-based and subjective success in the EEG-NF arm

**Figure 1:**
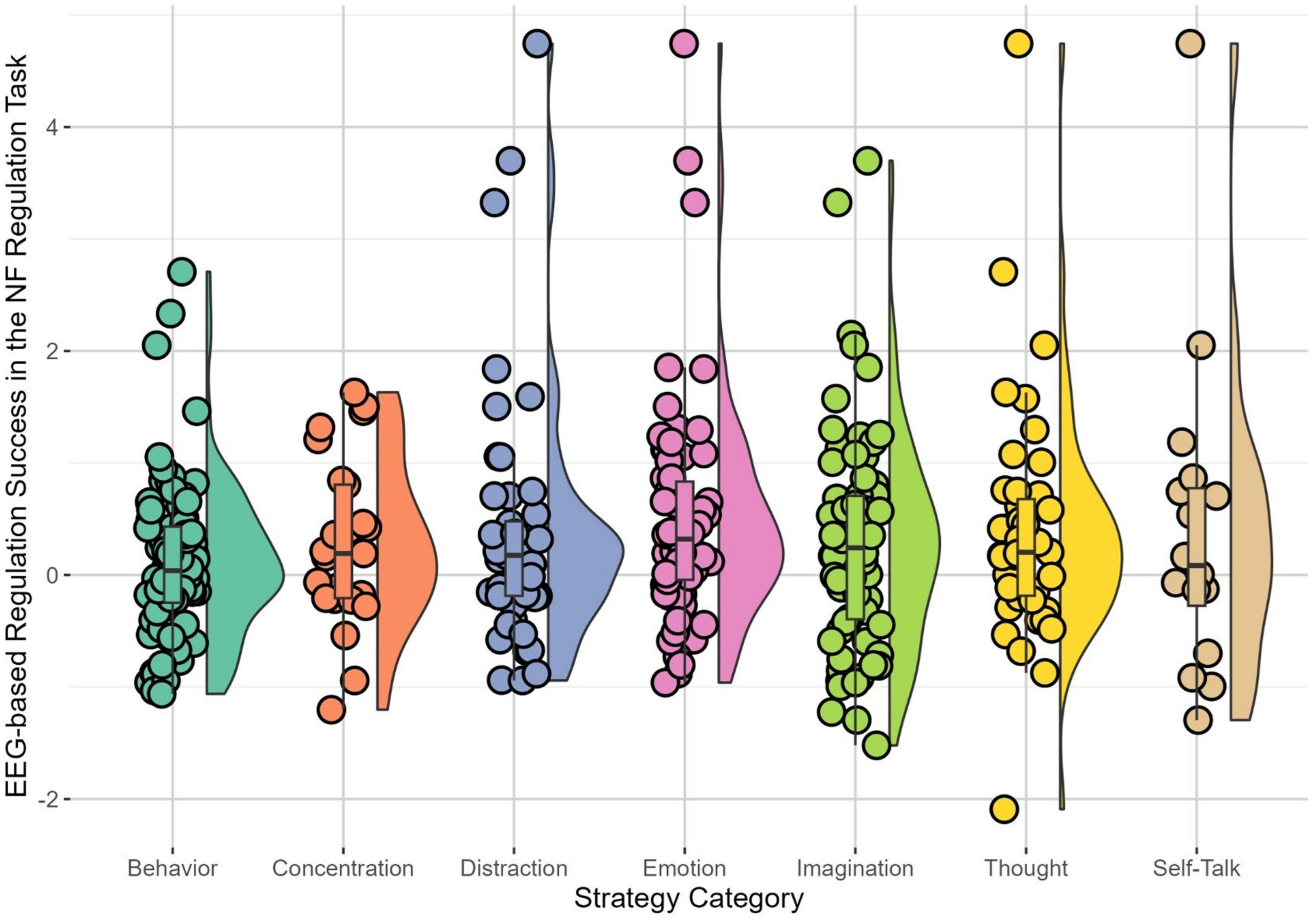
Patients’ (*n* = 23) brain-based success in the NF regulation task with immediate feedback for each strategy. Each dot showed the observed brain-based success, defined by the difference in high beta amplitude between regulation and baseline in the NF regulation task, when using the respective strategy. Horizontal lines indicated the median, whiskers indicated the first and third quartiles. The shape illustrates the distribution of EEG-based regulation success in the NF regulation task for the respective strategy. Results were averaged over sessions.

Only significant results were reported here; for further detail, see Supplementary Tables S3, S4. For the NF regulation task, “Imagination,” *B* = 0.13, *SE* = 0.05, *t* (236.40) = 2.51, *p* = 0.012, and “Behavior,” *B* = 0.17, *SE* = 0.05, *t* (238.40) = 3.46, *p* = 0.001, were significantly positive predictors for the difference of high beta amplitude in baseline versus regulation, indicating that patients in EEG-NF using these strategies had stronger success in downregulating high beta brain activity. In contrast, “Self-Talk,” *B* = −0.68, *SE* = 0.08, *t* (241.30) = −8.33, *p* < 0.001, and “Emotion,” *B* = −0.15, *SE* = 0.05, *t* (241.00) = −2.40, *p* = 0.017, emerged as significantly negative predictors for the difference of high beta amplitude in baseline versus NF regulation task, indicating that those who used these strategies during EEG-NF showed less success in downregulating high beta brain activity (Table 3, Figure 1).

**Table 3:** Final model for the prediction of EEG-NF success for the regulation task with immediate feedback by the strategies.

	*B*	SE	95% CI	*t*	*p*	*df*	Sum/Mean Sq	*F* ratio
Fixed effects								
Intercept	0.14	0.15	[−0.15, 0.43]	0.96	0.342			
Behavior	0.17	0.05	[0.07, 0.27]	3.46	0.001	*F* (1, 2383)	7.80	11.99
Concentration	0.13	0.07	[−0.02, −0.25]	1.70	0.090	*F* (1, 2431)	1.86	2.87
Distraction	−0.01	0.06	[−0.13, 0.12]	−0.07	0.944	*F* (1, 2185)	0.00	0.01
Emotion	−0.15	0.06	[−0.26, −0.03]	−2.40	0.017	*F* (1, 2410)	3.74	5.75
Imagination	0.13	0.05	[0.03, −0.23]	2.51	0.012	*F* (1, 2393)	4.08	6.28
Self-Talk	−0.68	0.08	[−0.85, −0.52]	−8.33	<0.001	*F* (1, 2412)	45.17	69.45
Thought	0.02	0.05	[−0.09, 0.12]	0.29	0.773	*F* (1, 2420)	0.05	0.08

Random effects	Variance	SD
Participant (intercept)	0.40	0.64
Session (intercept)	0.01	0.10

Model fit		
*R* ^2^	Marginal	Conditional
	0.05	0.42

EEG, electroencephalography; NF, neurofeedback. *p*-values for fixed effects calculated using Satterthwaite’s approximations. Confidence intervals have been calculated using the Wald method. All strategies were dummy-coded (0 = *Strategy was not applied in the respective session*, 1 = *Strategy was applied in the respective session*). Model equation: Neurofeedback difference value in baseline versus regulation ~ Concentration + Imagination + Self-Talk + Distraction + Thought + Emotion + Behavior + (1 | participant) + (1 | session). Due to its rare occurrence, the category “No Strategy” was not included in the analyses.

**Figure 2:**
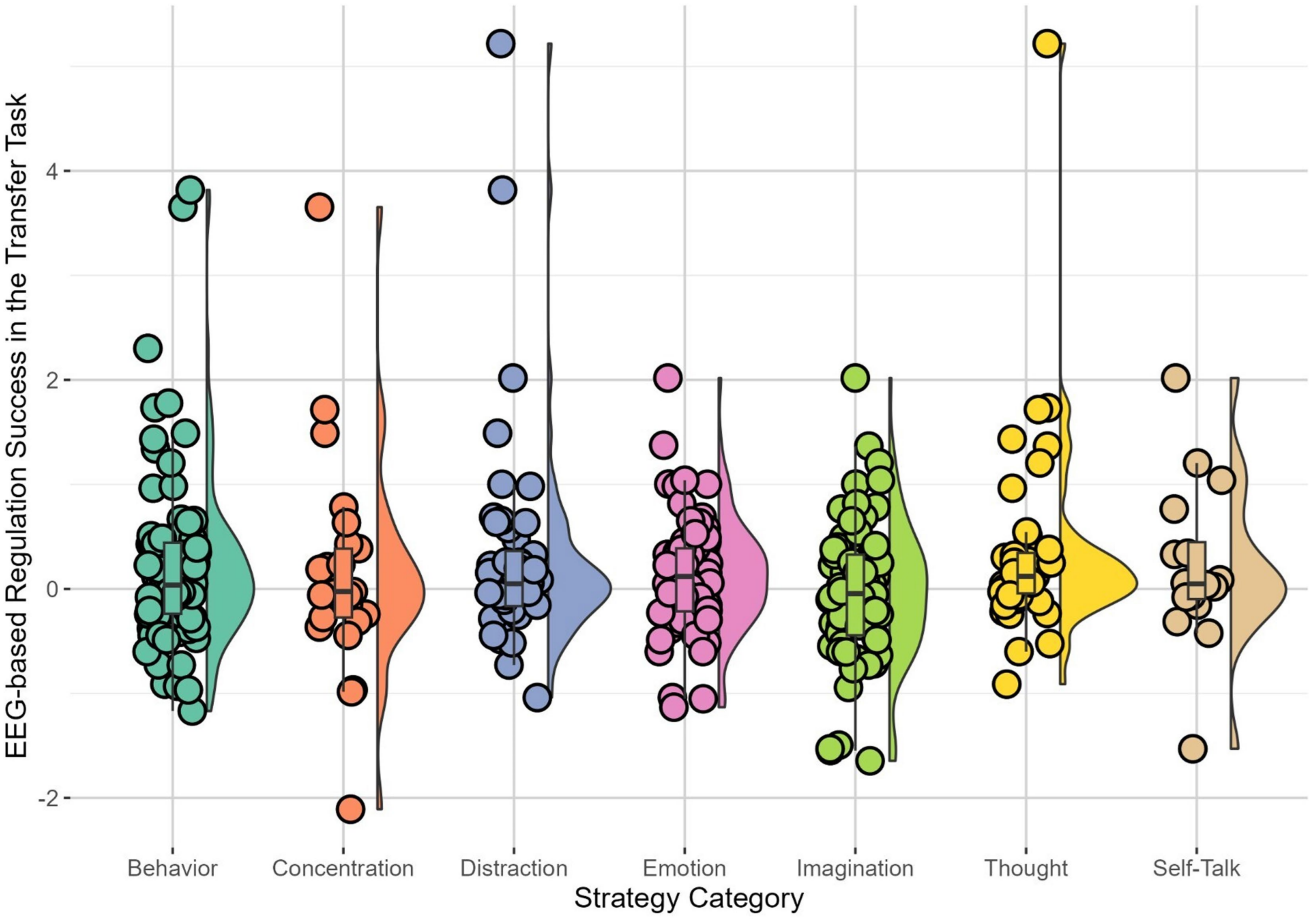
Patients’ (*n* = 23) brain-based success in the transfer task without immediate feedback for each strategy. Each dot showed the observed brain-based success, defined by the difference in high beta amplitude between regulation and baseline in the transfer task, when using the respective strategy. Horizontal lines indicated the median, whiskers indicated the first and third quartiles. The shape illustrates the distribution of EEG-based regulation success in the transfer task for the respective strategy. Results were averaged over sessions.

For the transfer task, patients in the EEG arm who used strategies of the category “Thought,” *B* = 0.21, *SE* = 0.07, *t* (1145.33) = 2.87, *p* = 0.004, showed stronger success in downregulating brain activity, whereas those who used “Emotion,” *B* = −0.21, *SE* = 0.08, *t* (915.06) = −2.56, *p* = 0.011, were less successful in downregulating brain activity (Table 4, Figure 2).

**Table 4:** Final model for the prediction of EEG-NF success for the transfer task without immediate feedback by the strategies.

	*B*	SE	95% CI	*t*	*p*	*df*	Sum/Mean Sq	*F* ratio
*Fixed Effects*								
Intercept	0.17	0.12	[−0.07, 0.41]	1.38	0.175			
Behavior	0.08	0.07	[−0.05, 0.22]	1.16	0.247	*F* (1, 1160)	0.87	1.34
Concentration	−0.01	0.09	[−0.19, 0.17]	−0.12	0.906	*F* (1, 1091)	0.00	0.02
Distraction	−0.09	0.09	[−0.26, 0.09]	−1.00	0.316	*F* (1, 727)	0.65	1.01
Emotion	−0.21	0.08	[−0.37, −0.04]	−2.56	0.011	*F* (1, 915)	4.25	6.55
Imagination	−0.08	0.07	[−0.22, 0.06]	−1.12	0.264	*F* (1, 1121)	0.80	1.25
Self-Talk	−0.05	0.11	[−0.27, −0.18]	−0.42	0.676	*F* (1, 1124)	0.11	0.17
Thought	0.21	0.07	[0.07, 0.36]	2.87	0.004	*F* (1, 1145)	5.34	8.24

Random effects	Variance	SD
Participant (intercept)	0.17	0.41
Session (intercept)	0.02	0.14

Model fit		
*R* ^2^	Marginal	Conditional
	0.04	0.25

EEG, electroencephalography; NF, neurofeedback. *p*-values for fixed effects calculated using Satterthwaite’s approximations. Confidence intervals have been calculated using the Wald method. All strategies were dummy-coded (0 = *Strategy was not applied in the respective session*, 1 = *Strategy was applied in the respective session*). Model equation: Transfer difference value in baseline versus regulation ~ Concentration + Imagination + Self-Talk + Distraction + Thought + Emotion + Behavior + (1 | participant) + (1 | session). Due to its rare occurrence, the category “No Strategy” was not included in the analyses.

**Figure 3:**
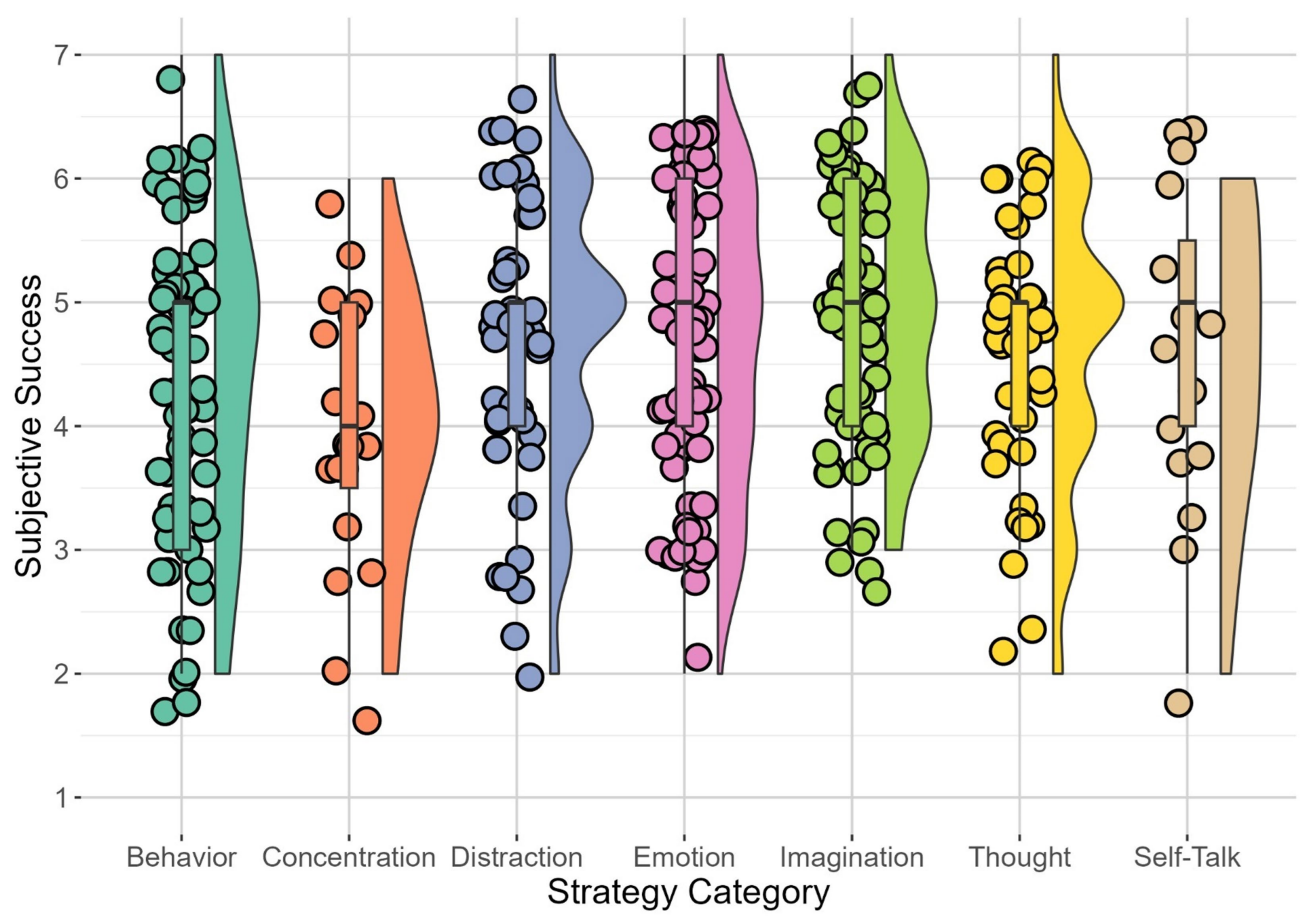
Patients’ (*n* = 23) perceived success for each strategy. Each dot showed the subjective success when using the respective strategy. Horizontal lines indicated the median, whiskers indicated the first and third quartiles. The shape illustrates the distribution of subjective success for the respective strategy. Results were averaged over sessions.

For subjective success, “Concentration” emerged as a significantly negative predictor, *B* = −0.84, *SE* = 0.29, *t* (167.59) = −2.92, *p* = 0.004, indicating that EEG-NF sessions during which “Concentration” was used were perceived as less successful by patients (Table 5, Figure 3).

**Table 5:** Final model for the prediction of subjective success after sessions by the strategies.

	*B*	SE	95% CI	*t*	*p*	*df*	Sum/Mean Sq	*F* ratio
Fixed effects								
Intercept	4.62	0.25	[4.13, 5.11]	18.14	0.175			
Behavior	−0.25	0.22	[−0.66, 0.17]	−1.16	0.242	*F* (1, 174)	1.46	1.36
Concentration	−0.85	0.29	[−1.40, −0.25]	−2.92	0.004	*F* (1, 168)	9.20	8.55
Distraction	0.07	0.24	[−0.38, 0.55]	0.30	0.767	*F* (1, 115)	0.10	0.08
Emotion	−0.10	0.23	[−0.53, 0.35]	−2.42	0.677	*F* (1, 126)	0.19	0.17
Imagination	0.40	0.22	[−0.03, 0.82]	1.79	0.076	*F* (1, 160)	3.44	3.20
Self-Talk	0.09	0.34	[−0.59, 0.73]	0.25	0.802	*F* (1, 160)	0.07	0.06
Thought	0.17	0.23	[0.27, 0.61]	0.76	0.451	*F* (1, 167)	0.61	0.57

Random effects	Variance	SD
Participant (intercept)	0.27	0.52

Model fit		
*R* ^2^	Marginal	Conditional
	0.10	0.28

*p*-values for fixed effects calculated using Satterthwaite’s approximations. Confidence intervals have been calculated using the Wald method. All strategies were dummy-coded (0 = *Strategy was not applied in the respective session*, 1 = *Strategy was applied in the respective session*). Model equation: Subjective success ~ Concentration + Imagination + Self-Talk + Distraction + Thought + Emotion + Behavior + (1 | participant). Due to its rare occurrence, the category “No Strategy” was not included in the analyses.

## 4. Discussion

This was the first study to investigate the use of mental strategies over 12 sessions of food-specific EEG- and rtfNIRS-NF in a clinical sample of patients with BED. Our study provided evidence for the diversity of mental strategies used to self-regulate brain activity, as reflected in eight strategy categories applied during NF treatment delivered in different imaging modalities (i.e., EEG-NF, rtfNIRS-NF). Patients mostly used “Imagination,” followed by “Behavior,” and “Self-Talk” strategies. Regarding brain-based success in the EEG-NF arm, the use of “Imagination,” “Behavior” and “Thought” strategies was associated with higher EEG-based regulation success in the NF regulation and transfer tasks, defined as the intended decrease in high beta relative to baseline activity. “Emotion” strategies were, in contrast, linked to lower EEG-based regulation success, and the use of “Concentration” strategies uniquely predicted lower subjective success, without an association with brain-based regulation success.

Our study showed that patients were able to identify a wide range of potential strategies in the absence of explicit instructions, using guided discovery only. We decided to consider patients from all randomization arms, benefitting from a larger database to derive a classification system with high validity and reliability (Erlingsson and Brysiewicz, 2013), also considering its application to future studies. The eight strategy categories that were extracted based on the written strategy reports by patients across all NF arms were comparable to categories found during previous qualitative analyzes of mental strategies reported in EEG-NF studies in healthy and patient samples (Kober et al., 2013, 2017; Hasslinger et al., 2020), including imaginative, behavioral, and emotional strategies. Considerable variability in strategy use between patients and sessions (see Supplementary Figure S1) suggested the continuous use of trial-and-error learning during the NF treatment.

Regarding the content of the mental strategies, patients across NF arms commonly applied task-oriented mental strategies encompassing the depicted food stimuli, such as imagining the food in a disgusting context in the most frequently used “Imagination” strategies. Altered food-related processing patterns specific to BED (Schmidt et al., 2016; Stojek et al., 2018; Chami et al., 2019) and BED with concurrent obesity (Paslakis et al., 2017) possibly fostered the use of disorder-specific food-related strategies in our sample with BED and overweight or obesity even in the absence of disorder-relevant stimuli (i.e., when only bars were presented on the screen during EEG-NF regulation trials). Previous clinical studies similarly reported the use of disorder-specific strategies, such as strategies targeting arousal regulation in ADHD with pertinent deficits in attention and impulsivity (Hasslinger et al., 2020); kinesthetic strategies in Parkinson’s disease with pertinent motion deficits (Bichsel et al., 2021); or food-related strategies in overweight and obesity (Spetter et al., 2017; Kohl et al., 2019). Accordingly, a study in healthy participants interpreted the lack of differences in the mental strategies used for various types of feedback signals (i.e., bars vs. worm avatars) in terms of the lacking match with participants’ preferences (Autenrieth et al., 2020). Importantly though, patient populations, as opposed to healthy participants, likely have a higher intrinsic motivation to perform better during NF treatment (Haugg et al., 2021) due to their goal to alleviate disease-specific symptoms (Blume et al., 2020). Indeed, the high burden of suffering of patients with BED relative to healthy participants may have increased their motivation to perform well during NF. This increased motivation may in turn have stimulated patients’ efforts to select a strategy targeting their pertinent BED symptoms. Alternatively, the “Imagination” strategies used by our patients may benefit their urges to eat binge foods through cognitive reappraisal, as binge foods were mostly imagined in ways that stressed their undesirability. This conjecture was supported by the demonstrated link between similar imaginative strategies encompassing reappraisal of the depicted foods and improved regulation of the urge to ingest craved foods in healthy participants (Giuliani et al., 2013). Regarding the use of mental strategies between groups, there are differences in the strategies used not only between EEG-NF and the rtfNIRS-NF group, but also between rtfNIRS-NF and delayed rtfNIRS-NF (see Supplementary Table S2). Thus, it is unlikely that neurocognitive mechanisms are the only factor with an influence on strategy use. Future research assessing the association between the target process and the emergence of mental strategies remains therefore imperative. Although studies highlighted the possible advantages of combining different modalities (Li et al., 2022), future research on the combination of these methods in eating disorders remains yet outstanding.

Turning to brain-based regulation success in the EEG-NF arm, high beta activity at its core has been identified to correlate with high performance and cognitive processing, but also rumination, overthinking, and worry (Demos, 2005). Regarding eating disorders, high beta activity was previously implicated as a neuronal marker for increased awareness of food cues in individuals with BED and with obesity (Blume et al., 2019; Hiluy et al., 2021). Increased fronto-central beta activity relative to individuals with normal weight correlated with eating disorder psychopathology in individuals with BED, while individuals with obesity also showed increased beta activity in the resting state. The consistently reported increased beta activity in fronto-temporal regions aligned with functional magnetic resonance imaging (fMRI)-based reports of alterations in the prefrontal cortex in BED and obesity (Donnelly et al., 2018; Mele et al., 2020). Most recently, studies on fNIRS in patients with BED and obesity suggest prefrontal hypoactivation in response to food stimuli when compared to normal weight controls (Rösch et al., 2021; Veit et al., 2021). Although these findings indicated dysfunctionalities in a brain network dedicated towards food-cue awareness and attentional bias, especially regarding the balance between inhibition and disinhibition, the exact interplay between EEG-based findings and the underlying brain activity assessed via fMRI or fNIRS has not yet been evaluated (Blume et al., 2019; Hiluy et al., 2021). Exploratory studies on the interplay of beta activity and neuronal activity linked beta activity to gamma-aminobutyric acid (GABA)-mediated inhibitory processes (Jensen et al., 2005; Yamawaki et al., 2008; Hall et al., 2011; Baumgarten et al., 2016). Most recently, a study on coordinated looking and reaching in non-human primates showed beta frequency to positively correlate with neural mechanisms responsible for inhibiting natural behavior such as coordinated movement (Hagan and Pesaran, 2022). Regarding the connection with BED and disordered eating, the modulation of GABA action has been mentioned as a potential target for future studies (Mele et al., 2020), but overall, research on the neural response to food stimuli is rare (Chae and Lee, 2023).

Regarding the association between strategy use and brain-based regulation success in the EEG-NF arm, “Emotion” and “Self-Talk” strategies were related to lower EEG-based regulation success. Verbal strategies, such as “Self-Talk” strategies, likely recruit substantial cognitive resources by requiring a level of awareness (Bastian et al., 2017) that hinders the integration of attentional and introspective processes (Alderson-Day and Fernyhough, 2015). Thus, “Emotion” and “Self-Talk” strategies may be regarded as explicit mental strategies which possibly induce cognitive overload (Kober et al., 2013) and thereby prevent the integration of various introspective processes necessary for NF success (Bagdasaryan & Le Van Quyen, 2013). Improved NF learning, defined as a positive correlation between session number and higher NF regulation success, was in contrast speculatively linked to a high level of automated and therefore subconscious control (Wood et al., 2014). Indeed, “Thought” strategies, subsuming cognitive attempts for free mind wandering which likely indicate a more automatic regulation, and “Behavior” strategies which presumably require little mental effort, emerged as positive predictors for greater EEG-based NF regulation success in healthy adults (Hardman et al., 1997; Kober et al., 2017). In summary, the search for an explicit mental strategy may induce cognitive overload (Khodakarami and Firoozabadi, 2020) and thereby hamper NF regulation success, whereas the absence of conscious strategy access potentially promoted internalization (Strehl, 2014).

Finally, recording subjective success via self-reports after each session may itself have kept patients motivated to reach their pre-defined goal of minimizing bar sizes during the 12 EEG-NF sessions (Abele and Spurk, 2009). The reporting of freely chosen strategies after each of the 12 NF sessions is unique to the present study, whereas previous studies only asked patients to report their strategies after the first and last of ten EEG-NF sessions (Kober et al., 2013). This thorough assessment of the mental strategies used possibly fostered patients’ continuous reflection on their own strategy use and thereby enhanced their motivation in the present study. Nevertheless, subjective success did not translate to changes in presumed underlying brain activity patterns in the EEG-NF arm, as the strategies that were most successful in terms of larger EEG-NF regulation success were not subjectively perceived as the most successful. This present finding stands in contrast to a previous study, showing that patients with epilepsy who successfully controlled their slow cortical potentials were able to sense their success after 30 of 35 EEG-NF sessions with an 8-week practice phase in between (Kotchoubey et al., 2002). Thus, more treatment sessions may be required before patients are able to correctly estimate the success of certain strategies. In light of limited evidence, the differences in objective, i.e., brain-based, and subjective regulation success and their association with the use of mental strategies should be explored in future studies.

## 5. Strengths and limitations

To our knowledge, no previous study examined the mental strategies used by patients with BED to self-regulate food-specific brain activity in NF treatment. We uniquely developed a new and fine-grained classification system via qualitative content analysis, based on written strategy reports provided after each session of EEG- or rtfNIRS-NF. Written self-report is the most common and feasible method to record mental strategies after NF (Nan et al., 2012; Kober et al., 2013, 2017). Assessment via interview is also possible (Hasslinger et al., 2020), however, the written strategy reports limited a potential interviewer bias on the participant (Davelaar et al., 2018) which likely affected previous coding systems (Hasslinger et al., 2020), including the induction of a good-subject bias (Nichols and Maner, 2008). Importantly, every single strategy report was considered carefully, and categories only emerged after several steps of analysis and consensus within the research team. Previous strategy classification approaches, on the contrary, were based on the experimenter’s subjectively determined categories (Nan et al., 2012; Kober et al., 2013), or made use of existing classification systems developed in healthy samples (Kober et al., 2017; Autenrieth et al., 2020). In addition, instead of solely relying on consensus between the raters (Hasslinger et al., 2020), raters in the present study independently assigned strategies to the preformed categories before reaching a consensus. High inter-rater reliability supported the validity of our new classification system, making it well-suited to categorize the strategies used by patients with BED for food-specific NF self-regulation. A further strength of this study was its analytical approach, ensuring robustness in small samples (Luke, 2017) via the use of linear mixed models, while comprehensively including all random effects in accordance with recommendations (Barr et al., 2013) in order to account for non-independent data (Brauer and Curtin, 2018).

A possible limitation was that we could not trace back strategies to single trials, as all strategies used during one session were reported at the end of the NF session. Consequently, the percentage of time a strategy was used during one session could not be determined, resulting in a potential over- or underestimation of the strategies’ relevance. It remains unknown whether patients used a combination of strategies rather than a single strategy to modulate brain activity during each trial. This assessment of strategies via self-report after the end of a NF session may have introduced order effects in the form of primacy or recency effects (i.e., patients may have remembered their first or last mental strategies; Murdock, 1962) and errors in reporting. In addition, patients may not have remembered all strategies they used during the 12 NF trials. While strategy assessment via interview would also have been subject to both of these effects and despite the previously discussed pertinent advantages of recording subjective success via self-report, we cannot rule out that other strategies may have been spontaneously verbalized if strategies were assessed through an interview (e.g., Hasslinger et al., 2020). In addition, qualitative data analysis is inherently subject to experimenter’s subjective decisions, although the consensus discussions and the assessment of inter-rater reliability aimed to increase generalizability of the present findings. In this context, it deserves mention that the current classification of patients’ mental strategies was based on the full sample of patients who underwent the NF procedure, irrespective of the processes targeted through each specific imaging modality. Importantly though, the emergence of strategies may depend on the underlying process even if the provided feedback was similar across the processes (through individually appetitive food cues in our study). Future studies are therefore needed to disentangle the degree to which the emergence of strategies is affected by the processes targeted through the imaging modality, even if feedback is given through a similar brain-computer interface. Another limitation was the implicit data analytic assumption that the association between strategy use and brain-based or subjective regulation success was the same across participant. In fact, previous research emphasized that strategies which were successful on the group level may not be successful on the participant level and vice versa (Barth et al., 2016). Regarding the sample, we cannot rule out for that patients’ comorbidities and subsequently, their medication, had an influence on the selection and use of mental strategies. However, since BED is an affliction with a high potential for comorbid somatic conditions (Udo and Grilo, 2019), this was taken into account during patient selection: The use of medication such as antidiabetics, which might have an influence on eating behavior, weight and/or executive function and therefore, on the use of mental strategies was only permissible after a minimum duration and if the dosage had been stable for several months (see Supplementary material of Hilbert et al., 2023), to minimize effects on the use of mental strategies. Future explorative analyzes should consider the potential influence of patients’ characteristics, in particular the role of concurrent weight status and sex on mental strategy use and on the link between subjective and brain-based success. Lastly, the current study focused on patients randomized to EEG-NF, with likely differential links between strategy use and EEG- versus rtfNIRS-based regulation success reported elsewhere (Rösch et al. (2023) “Mechanisms underlying fNIRS-neurofeedback over the prefrontal cortex for participants with binge-eating disorder”). Future studies may harness the pertinent advantages of both imagining modalities in combined applications to provide more comprehensive information on the functional activity of the brain through the concurrent assessment of neuronal electricity activity (EEG-NF) and metabolic response (fNIRS-NF; Li et al., 2022).

## 6. Conclusion

In summary, our study extended previous research on the use of mental strategies throughout NF treatment by firstly deriving a classification system for mental strategies used in response to individually appetitive food cues in adult patients with BED. The classification system provided valuable information about efficient strategies targeting BED-specific processing of individually appetitive food stimuli. Based on our findings, future clinical NF studies could examine whether explicit instructions of imaginative strategies, which were linked to better EEG regulation success, were likewise associated with favorable clinical outcomes (Herwig et al., 2019). Strategies which were linked to greater brain-based regulation success were more frequently used than the strategies that prove herein less successful, in line with the presumed operant conditioning principle underlying NF learning. Successful strategies were likely internalized due to the continuous reinforcement during the NF sessions, and then shown in an automatic manner not requiring conscious attention. Importantly though, the differential link between the mental strategies used and objective and subjective success warrants replication in future research, which should ideally also assess whether brain-based and subjective success translate to clinical symptom improvements.

## Data availability statement

The raw data supporting the conclusions of this article will be made available upon request to the corresponding author.

## Ethics statement

The studies involving humans were approved by Ethics Committee of the University of Leipzig (474-ek). The studies were conducted in accordance with the local legislation and institutional requirements. The participants provided their written informed consent to participate in this study.

## Author contributions

AH and RS: conceptualization, methodology, software, and project administration. SR and RS: validation and investigation. JW and SR: formal analysis and writing – original draft preparation. AH: resources, supervision, and funding acquisition. JW, RS, and SR: data curation. AH, JW, RS, and SR: writing – review and editing. SR: visualization. All authors contributed to the article and approved the submitted version.
