# Supplementary material for: Neurofeedback strategies in binge-eating disorder as predictors of EEG-neurofeedback regulation success
Source: Front Hum Neurosci. 2023 Oct 30;17:1234085. doi: 10.3389/fnhum.2023.1234085 (PMC10645064; doi:10.3389/fnhum.2023.1234085)
Supplement: Supplementary file 1 [file Data_Sheet_1.docx]

Supplementary Material for

Neurofeedback Strategies in Binge-Eating Disorder as Predictors of EEG-Neurofeedback Regulation Success

Jytte Wimmer, Sarah Alica Rösch, Ricarda Schmidt, Anja Hilbert

# Supplementary Methods

## Study Design

This study was conducted in the context of the randomized-controlled trial “Near Infrared Spectroscopy Neurofeedback for Binge-Eating Disorder” (NIRSBED; Hilbert et al. 2023; DRKS00014752, www.drks.de). Inclusion criteria for this study encompassed full syndrome binge-eating disorder (BED) or BED of low frequency and/or limited duration (American Psychiatric Association, 2013) assessed by the German version of the Eating Disorder Examination interview (Hilbert & Tuschen-Caffier, 2016), BMI 25.00-40.00 kg/m², age ≥ 18 years, sufficient German language skills, and feasible commute to the study site. Exclusion criteria were serious somatic conditions (e.g., head injury, neurological disorder), serious mental disorder (e.g., psychotic disorder, suicidality), impediment in language, hearing, or vision that might affect testing, bariatric surgery (previous or planned), medication that might impact weight or executive functions, ongoing psychotherapy, and pregnancy or lactation. Patients were also excluded if they had medication with an impact on weight or executive functions, unless medication was stable for at least 2 months or for at least 6 months for diabetes drugs. Patients were recruited from an in-house research database, information events for a behavioral weight loss treatment program at the Obesity Outpatient Unit at the Leipzig University Medical Center, and from the population (e.g., Internet advertisements). After a detailed explanation of the study procedures, written informed consent was obtained from all patients prior to study participation.

## Patients

After inclusion, patients were randomly assigned to electroencephalography (EEG)- or real-time functional near-infrared spectroscopy (rtfNIRS)-neurofeedback (NF) or delayed rtfNIRS-NF, receiving rtfNIRS-NF after a waiting period of eight weeks. Previous research indicated that successful regulation during the first NF treatment session may happen coincidentally and that patients need the first NF treatment session to accustom themselves to the instructions and restrictions of the NF modality (Drechsler et al., 2007; Weber et al., 2020). Therefore, for inclusion in this study, ≥ 2 NF sessions were required, resulting in a sample of *n* = 63 patients who were analyzed for the present study.

## Food Picture Selection

Prior to NF treatment, patients were shown 70 food pictures from the Food-pics database (Blechert et al., 2014), inclusive of 25 pictures of savory, 22 pictures of sweet, 11 pictures of salty meals and 12 pictures of fruit and vegetables. Patients marked whether the depicted food was part of a binge-eating episode (0 = *no*, 1 = *yes*) during the past four weeks and indicated their craving for the depicted food picture on a 100-point continuous scale ranging from 0 = *not at all* to 100 = *very strong*. The thirteen (or twelve in the rtfNIRS groups; see subsequent sections) pictures that were part of a binge-eating episode and had the highest craving scores were selected as individually appetitive food pictures for the six following NF sessions. To avoid habituation and to promote generalization of effects, the same procedure was repeated prior to the seventh session and new pictures were selected to be presented in sessions 7-12.

## EEG-NF

A NEURO PRAX® EEG-full-band DC-EEG Bio- and Neurofeedback-System by Neurocare (THERA PRAX® neuroConn GmbH, Ilmenau, Germany) was used for EEG-NF. The EEG was herein derived from four Ag/AgCl electrodes from Fz, Cz, Fc1, and Fc2 as training sites in reference to the mastoids with a sampling rate of 256 samples per second. For filtering, a band-pass filter of 0.53-70 Hz and a notch filter of 50 Hz were used; EOG and heart rate were assessed. Impedances were kept below 10 GΩ.

Since the spatial resolution of EEG is limited to about 5-9 cm (Babiloni et al., 2001; Nunez et al., 1994) it is not possible to dissociate what happens in different subdivisions of the fronto-central region (a target area of altered brain function in patients with BED and obesity; Karhunen et al. 2000; Tammela et al. 2010; Blume, Schmidt, and Hilbert 2019). Higher spatial resolution is possible, but requires a much larger number of electrodes (Kwon et al., 2019) not deemed necessary for our purpose, since our set-up of four electrodes (Fz, Cz, Fc1, Fc2) was based on a validated protocol for BED (Blume et al. 2022).

Session procedures were similar to a previous EEG-NF study in our laboratory (Blume et al., 2022). Each session consisted of a 180 s baseline measurement and a NF and a transfer task and lasted for approximately 60 min. During the baseline measurement, which served as an individual threshold for the subsequent regulation trials of each task, patients were instructed to keep their eyes open and look at the fixation cross in the middle of the screen. The first baseline recording was followed by 12 NF regulation trials consisting of alternating phases of regulation and food presentation, starting with a regulation phase without prior food presentation. During food regulation trials, patients were instructed to decrease the bars below the yellow line, which represented their baseline for 60 s, while keeping their eyes open. Patients were informed that no pre-set way to regulate their brain activity existed. Instead, they should explore which strategy worked best for them during regulation trials, while keeping their eyes open and their bodies as still as possible. During food presentation trials, patients were instructed to imagine the individually selected food pictures, which were presented by the trainer, as vividly as possible. After 25 s had passed, the picture disappeared and two bars were depicted on the screen, mirroring current high beta activity and muscle activity. After the 12 trials, an adaptation phase followed, which consisted of another baseline recording. The following six transfer blocks consisted of alternating trials of food presentation and regulation. The instruction in transfer trials was identical with the NF trials, but patients were not provided real-time feedback during these transfer regulation trials. They were instead displayed the percentage of time they managed to keep their high beta activity below the threshold at the end of each trial. During sessions, the NF therapist manually noted the mean amplitude of the baseline, NF regulation task and transfer regulation task for high beta activity and muscle activity in µV and the number of successful trials per NF regulation task and transfer regulation task, defined as trials during which high beta activity was below baseline activity.

## Inter-Rater Reliability

After the second rater (SR) was introduced to the first rater’s (JW) classification key (see Results section), she rated whether each category occurred in the individual reports (levels: *yes*, *no*), using as many categories as necessary to describe their contents in an open-ended multiple-choice format (i.e., multiple categories per strategy report were possible and common). Both raters generated a 227 (18 patients * 12 sessions + 1 participant * 11 sessions) × 24 (number of sub-categories) matrix of binary values. In case of different category assignment of both raters to identical categories, Kappa estimates were lowered for both the category assigned by Rater 1 and the category assigned by Rater 2, resulting in Cohen’s Kappa as a conservative estimate.

## Outlier Exclusion

Patients’ single trials in the NF regulation task or transfer regulation tasks were excluded from the analyses in case of *z*-scores ≥ 3 relative to the participant’s individual mean high beta amplitude in the respective task across sessions (NF and transfer regulation and baseline; Chow et al. 2017; Hoedlmoser 2007; Hoedlmoser et al. 2008). Using this procedure, a total of *n* = 129/4151 trials in the regulation tasks were excluded (3.11 %), specifically, *n* = 83/2771 trials in the NF regulation tasks (3.00 %) and *n* = 46/1380 trials in the transfer regulation task (3.33 %). For the baseline condition, no trial was excluded.

## Statistical Data Analysis

Mixed models were applied due to their advantages over classical analysis of variance, such as not assuming independence among observations (and thereby accounting for variability within and across patients and sessions) and greater robustness for unbalanced designs and missing data (Bates et al., 2015; Laird & Ware, 1982; Meteyard & Davies, 2020). All data were examined for normality and sphericity and the assumption of normally distributed residuals was checked via a visual inspection of the QQ-plot. We followed a backward selection approach, starting with maximal models (Barr et al., 2013) to control for type I error rate inflation through inclusion of all random intercepts and slopes justified by the experimental design. Random intercepts at the participant, session, and trial level accounted for within-participant correlations. In accordance with recommendations (Brauer & Curtin, 2018), separate models for each of the random intercepts were carried out if convergence issues occurred, applying the *F*_1_ x *F*_2_ logic. Random intercepts were retained if model comparisons (via χ²-tests) indicated significant differences between the log-likelihood (based on the Akaike and Bayesian Information Criteria) of a model that contained the random effect and a model that did not (as compared with ANOVA; *p* <.05; see supplementary Table S4). The Akaike Information Criterion was favored over the Bayesian Information Criterion due to the exploratory nature of our study (Aho et al., 2014). Regression coefficients and *R*² of the fixed effects of these mixed models were calculated with the function r.squaredGLMM from the package MuMin (Barton, 2020).

## Explorative Analyses

After having derived the relative frequency of each strategy (i.e., the occurrence of a strategy category relative to the total number of strategy reports), these relative frequencies of the reported mental strategies were statistically compared between groups (EEG versus rtfNIRS versus delayed rtfNIRS-NF) using the Pearson’s Χ²-Test statistic (Kober et al., 2020), following a request during the revision process.

An explorative analysis was carried out to assess the relationship between self-reported and brain-based success. To this end, two models were calculated in which self-reported success after each session was regressed on the mean high beta difference (across trials) in the baseline versus regulation condition (with higher values indicating greater brain-based success) in the regulation and in the transfer task.

- 1. **Example of Qualitative Analysis Process**

The strategy report: *“Imagined the food to be not tasty but disgusting”* was condensed to reflect the core meaning, resulting in the content unit: “*Imagination food disgusting not tasty*.” The code of this condensed meaning unit: “*Imagination inducing disgust”* was assigned to the subcategory: “*Imagination of food negative,”* which fell under the broader category: “*Imagination*.*”* These categories and subcategories were adjusted and underwent a dynamic process of definition and redefinition as categorizing progressed.

## Categories for Strategies

***Behavior*** described strategies grounded in a certain behavior. This category consisted of either mental or physical relaxation (*Relaxation*) strategies attempting to modify the breath (*Breathing*), and strategies targeting physical activity (*Physical activity*).

***Concentration*** described any strategy used to concentrate or focus on something. Three subcategories were formed: *Concentration on task,* *Concentration on self/body, Concentration on surroundings*.

***Distraction*** encompassed planning the day or thinking of set routines (*Distraction everyday life*), included strategies of auditive imagination, sometimes with a visual component (*Distraction music/movies*), or revolved around counting or listing names (*Distraction words/numbers*).

***Emotions*** was a category for strategies used to evoke either positive emotions (*Inducing positive emotions*), for instance through thoughts of places, activities or loved ones, or negative emotions (*Inducing negative emotions*), for example, through thoughts of rejection or great pain.

***Imagination*** included strategies promoting the mental emergence of pictures, feelings, or notions. The first subcategory was *Imagination of changed visual perception*, for example, adding things to visual perceptions or imagining things that were not there. Patients who used strategies in the *Imagination of movement* subcategory imagined moving their bodies, often through exercise. In the category *Imagination of food defense*, patients imagined fending off the food pictures. Concerning food, three additional *Imagination* categories were extracted: *Imagination of food neutral* encompassed emotionally neutral imaginations of the depicted food, for example, its preparation. *Imagination of food positive* were strategies centered on relearning food enjoyment as well as imagining a healthy alternative. Patients using strategies from the *Imagination of food negative* category, on the other hand, mentally made the presented visual food stimuli as unattractive as possible to themselves, for instance by imagining the depicted food getting moldy. Lastly, *Imagination* also included the categories *Imagination positive for self-motivation* and *Imagination negative for self-motivation*. The former category included strategies focused on imagining the possible positive consequences in case of a success of the NF treatment, whereas the latter emphasized the negative consequences of an unchanged eating behavior.

***Self-Talk*** included strategies where patients held an inner dialogue. *Verbal avoidance of overeating* comprised sentences about or directed at the foods, while *Verbal directed at self* consisted mostly of self-reinforcing sentences.

***Thoughts*** included strategies of patients that either made an effort to not think of anything specific (*Thoughts of nothing specific*) or explicitly avoided all thoughts of food (*Thoughts of food avoidance*).

***No Strategy*** was a category for those patients who reported not to have used any strategy.

# Supplementary Results

## Effects of Regulation Success on Perceived Success

An exploratory analysis revealed that, for the regulation task (see Supplementary Figure S4), the difference between baseline and regulation was a significant and negative predictor of perceived success, *B* = -0.07, *SE* = 0.02, *t* = -2.96, *p* = .003, indicating that those with a smaller difference between baseline and regulation (i.e., who succeeded less in downregulating beta activity) perceived themselves as more successful. In contrast, for the transfer task (see Supplementary Figure S4), the difference between baseline and regulation significantly positively predicted self-reported success, *B* = 0.37, *SE* = 0.03, *t* = 11.24, *p* <.001, indicating that those with a higher difference (i.e., who managed to downregulate beta activity) perceived themselves as more successful.

# Supplementary Figures and Tables

## Supplementary Figures

**Supplementary Figure S1.** Frequency of strategy categories present in reports per total reports of patients undergoing EEG-NF (*n* = 23) displayed by session.


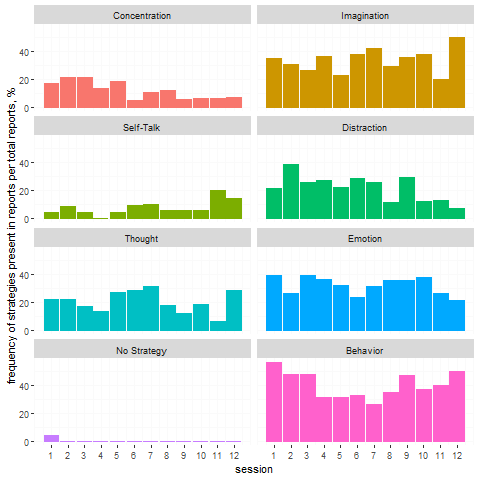


**Note.** EEG = electroencephalography, NF = neurofeedback. The x-axis depicts the respective session, the y-axis depicts the frequency of a strategy category in the reports per total reports of the respective session. 100% consequently indicates that that all patients used the respective strategy in the respective session. Due to the open-ended multiple-choice format, one and the same strategy report could be assigned to multiple categories.

**Supplementary Figure S2.** Patients’ brain-based success over the time course of 12 EEG-NF sessions.


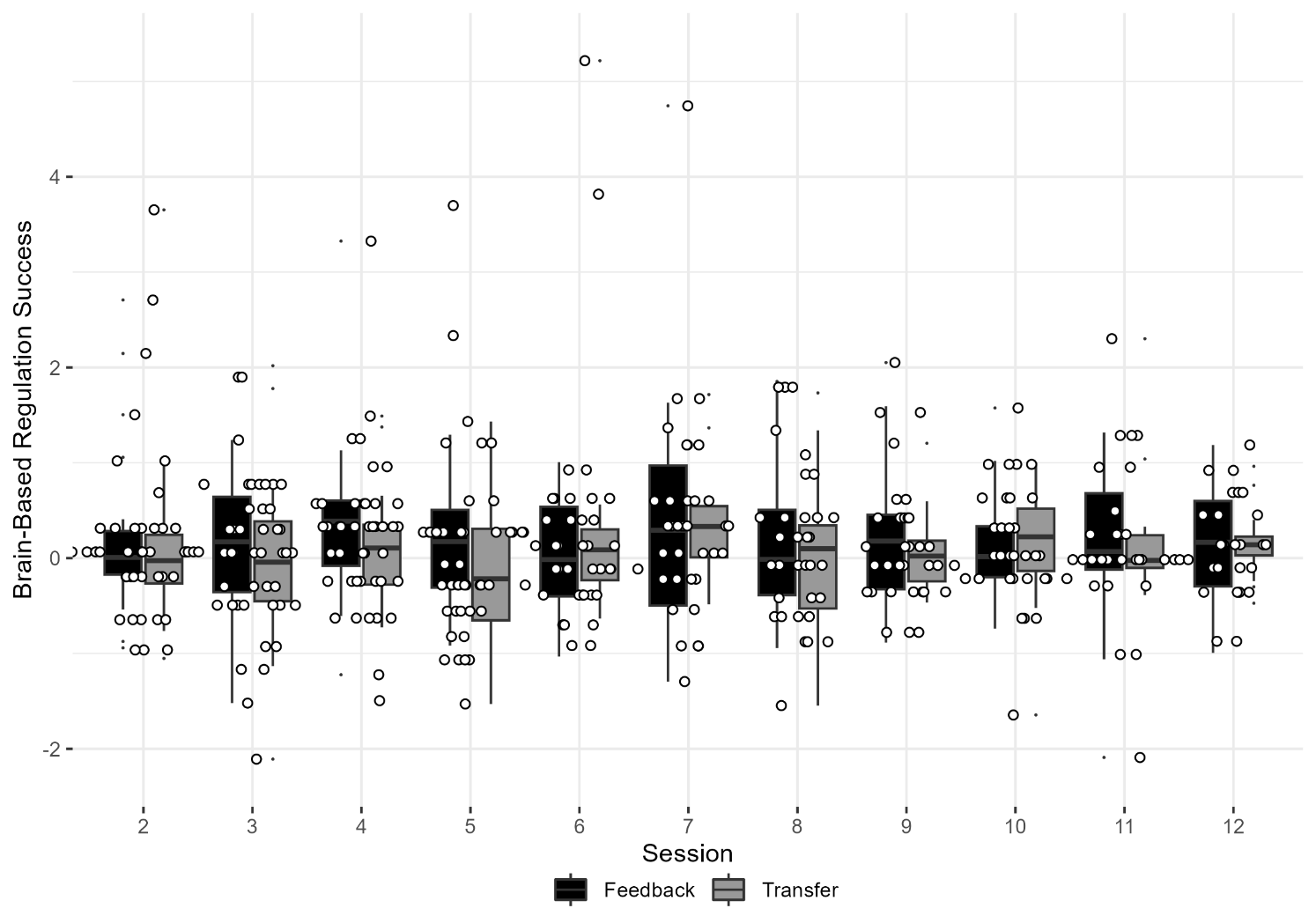


**Note.** *n* = 23. EEG = electroencephalography, NF = neurofeedback. The x-axis depicts the respective session, the y-axis depicts the EEG-based regulation success, defined by the difference between regulation and baseline in the respective session and task. Each dot showed the observed brain-based success for each patient. Horizontal lines indicated the median, whiskers indicated the first and third quartiles.

**Supplementary Figure S3.** Patients’ subjective success over the time course of EEG-NF sessions.

**
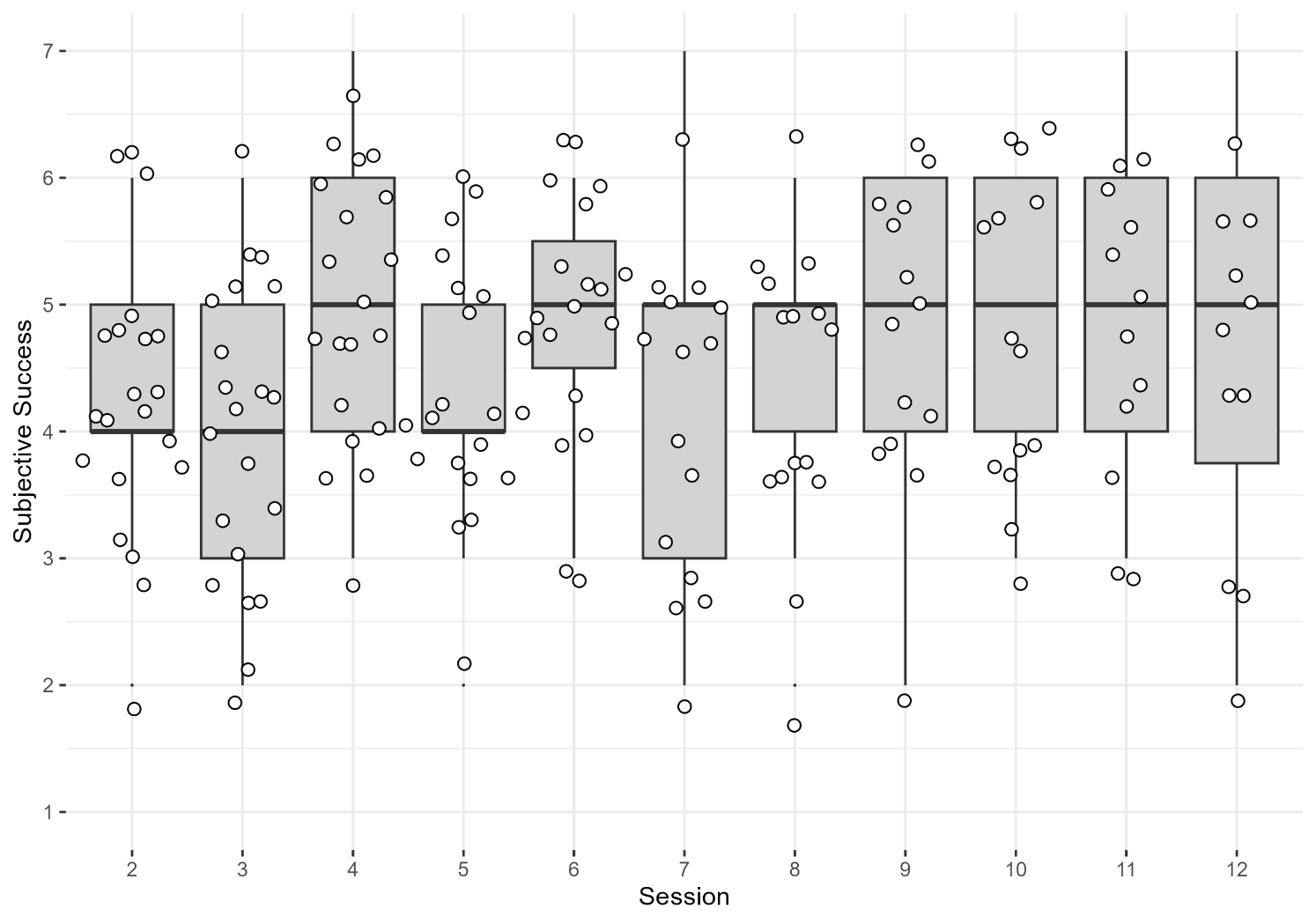
 Note.** *n* = 23. EEG = electroencephalography, NF = neurofeedback. The x-axis depicts the respective session, the y-axis depicts the subjective success. Each dot showed the observed subjective success for a single patient in the EEG-NF group for the respective session. Horizontal lines indicated the median, whiskers indicated the first and third quartiles.

**Supplementary Figure S4.** Relationship between subjective and brain-based regulation success.

**
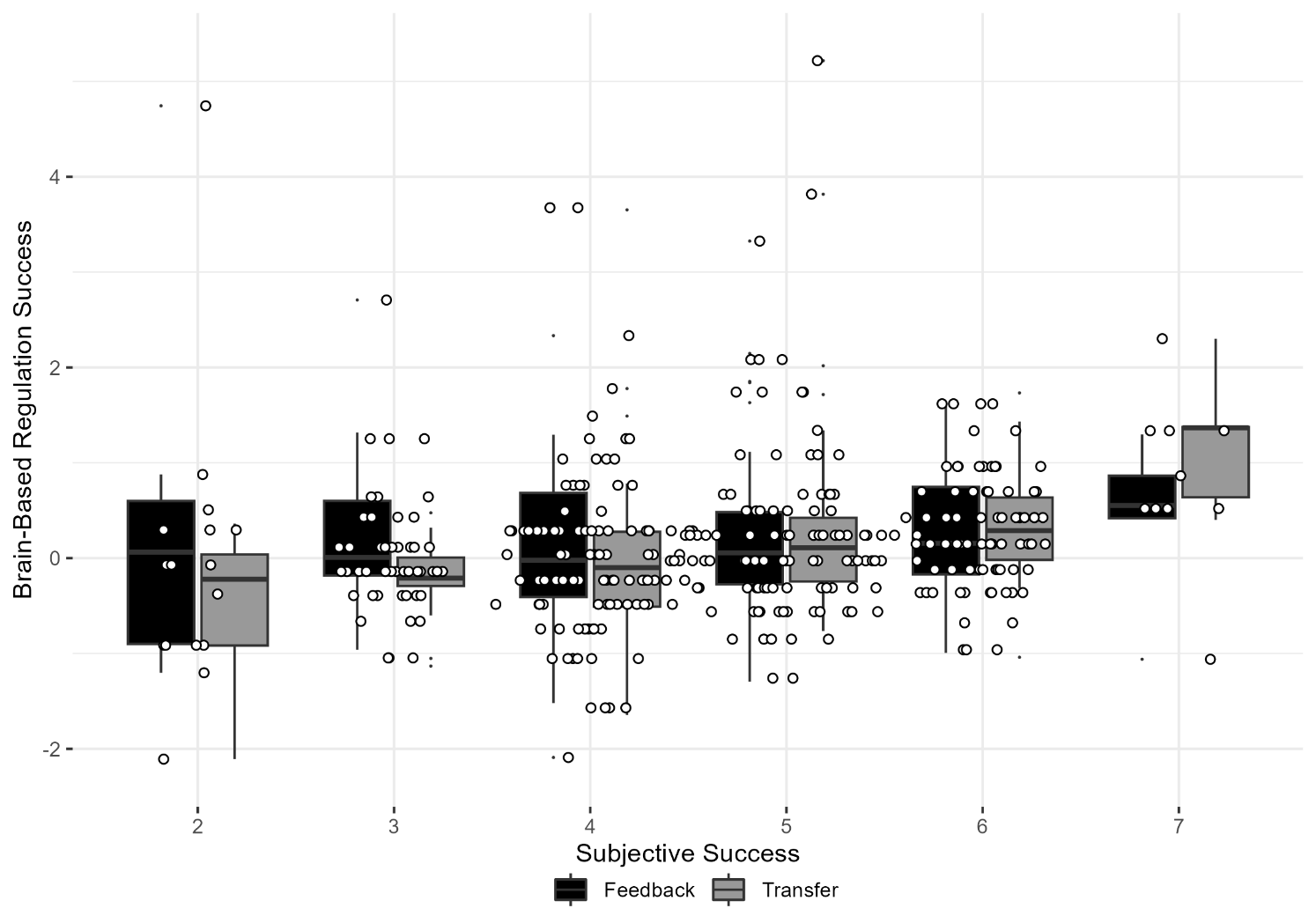
**

**Note.** *n* = 23. The x-axis depicts the subjective success, the y-axis depicts the brain-based regulation success in the respective task. Results were averaged over sessions. Each dot showed the observed brain-based success for a single patient in the EEG-NF group for the respectively reported subjective success. Horizontal lines indicated the median, whiskers indicated the first and third quartiles.

## Supplementary Tables

**Supplementary Table S1.** Examples, inter-rater reliability, and frequency of use for each category and subcategory across treatment arms (i.e., EEG-NF, rtfNIRS-NF, and delayed rtfNIRS-NF).

| **Category** | **Example** | **Cohen’s kappa coefficient** | ***n*** | **% used** |
| --- | --- | --- | --- | --- |
| ***Behavior*** |  | .99 | 190 | 27.82 % |
| *Relaxation*  *Breathing*  *Physical activity* | I tried to relax  Paid attention to breathing steadily  Pressed tongue against upper jaw |  | 101  95  31 | 14.79 %  13.32 %  5.12 % |
| ***Concentration*** |  | .69 | 118 | 17.80 % |
| *Concentration on task* | Directing all [mental] energy toward the task |  | 56 | 8.20 % |
| *Concentration on self/body* | Feeling [the] feet firmly on the ground |  | 26 | 3.81 % |
| *Concentration on surroundings* | Focus on the text on the monitor |  | 40 | 5.86 % |
|  |  |  |  |  |
| ***Distraction*** |  | .67 | 91 | 13.32 % |
| *Distraction everyday life*  *Distraction music/movies*  *Distraction words/numbers* | To-do lists in my head  Train ride with specific people that always get in  Played/sang a song in my head, played a movie trailer in my head  Counted forwards and backwards |  | 39  45  20 | 5.71 %  6.59 %  2.93 % |
| ***Emotion*** |  | .89 | 115 | 16.84 % |
| *Inducing positive emotions*  *Inducing negative emotions* | Thoughts of nice situations with grandchild  Negative thoughts, rejection |  | 101  16 | 14.79 %  2.34 % |
| ***Imagination*** |  | .69 | 289 | 42.31 % |
| *Imagination of changed visual perception* | Looking for people in the meals |  | 33 | 4.83 % |
| *Imagination of movement* | Sense of exercising/jogging |  | 20 | 2.93 % |
| *Imagination of food defense* | Pushing the pictures away in my thoughts |  | 47 | 6.88 % |
| *Imagination of food neutral* | Imagined how the food is made |  | 35 | 5.12 % |
| *Imagination of food positive* | No prohibitions, enjoying small portions |  | 77 | 11.27 % |
| *Imagination of food negative* | Concerning the sweets, I imagined all that sugar [in the depicted food] and pretended it was poison |  | 52 | 7.61 % |
| *Imagination for positive self-motivation* | Imagined the new, healthy life I would have after weight reduction |  | 80 | 11.71 % |
| *Imagination for negative self-motivation* | I saw myself as a very fat man |  | 26 | 3.81 % |
|  |  |  |  |  |
| ***Self-Talk*** |  | .75 | 177 | 25.92 % |
| *Verbal avoidance of overeating* | When [shown] unhealthy food, I thought “Go away!” |  | 145 | 21.23 % |
| *Verbal directed at self* | Sentences like “I am in control!”, “I decide, not the food” |  | 56 | 8.20 % |
|  |  |  |  |  |
| ***Thought*** |  | .84 | 102 | 14.93 % |
| *Thoughts of nothing specific* | Tried to empty head |  | 75 | 10.98 % |
| *Thoughts of food avoidance* | Seeing the food without thinking about it |  | 30 | 4.39 % |
|  |  |  |  |  |
| ***No Strategy*** | Tried several things  Hard to explain | .00^a^ | 13 | 1.32 % |

**Note.** *N* = 683. EEG = electroencephalography, NF = neurofeedback, rtfNIRS = real-time functional near-infrared spectroscopy.

Percentage of subcategories refer to the number of reports of subcategory use relative to the total number of reports, but may not sum up to the number of overarching categories because overarching category was only assigned in a dichotomous format (yes/no)

^a^The low number of participants using “No Strategy” potentially undermined the validity of inter-rater reliability analyses.

**Supplementary Table S2.** Inter-rater reliability and frequency of strategies in reports per total reports in each group.

| **Category** | **Cohen’s Kappa Coefficient (*n* = 19)** | **% used** |  |  |  |  |  |  |
| --- | --- | --- | --- | --- | --- | --- | --- | --- |
|  |  | **Across groups (*n* = 63)** | **EEG (*n* = 23)** | **rtfNIRS (*n* = 22)** | **delayed rtfNIRS-NF (*n* = 18)** | **Χ²(2, *N* = 682)** | ***p*** | **Post-hoc tests** |
| Concentration | .69 | 17.30 % | 12.93 % | 18.11 % | 21.26 % | 3.89 | .143 |  |
| Imagination | .69 | 42.38 % | 33.19 % | 46.91 % | 47.34 % | 5.09 | .078 |  |
| Verbal | .75 | 25.81 % | 7.33 % | 27.16 % | 44.93 % | 48.97 | <.001 | delayed rtfNIRS > rtfNIRS > EEG |
| Distraction | .67 | 13.34 % | 23.28 % | 9.47 % | 6.76 % | 22.71 | <.001 | EEG > rtfNIRS, delayed rtfNIRS |
| Thoughts | .84 | 14.96 % | 20.69 % | 8.64 % | 15.94 % | 10.30 | .006 | EEG > rtfNIRS |
| No strategy | .00 | 1.61 % | 0.43 % | 4.94 % | 0.00 % | 17.75 | <.001 |  |
| Emotions | .89 | 16.86 % | 32.33 % | 6.58 % | 11.59 % | 42.21 | <.001 | EEG > delayed rtfNIRS > rtfNIRS |
| Behavioral | .99 | 27.86 % | 40.52 % | 26.75 % | 14.98 % | 20.31 | <.001 | EEG > rtfNIRS > delayed rtfNIRS |

**Note**. EEG: electroencephalography group, rtfNIRS: real-time functional near-infrared spectroscopy group.

**Supplementary Table S3.** Checklist for best practice guidance for reporting LMMs (Meteyard and Davies 2020).

| **Issue** | **Recommendation** | **Reported in section** |
| --- | --- | --- |
| Software | Report the software and version of software used for modelling | 2.4 Statistical Analysis |
| Power analysis | Report any a-priori power analyses, including effect sizes for fixed effects and variances for random effects. | Not applicable |
| Assumptions of LMM | Report what data cleaning has been completed, outlier/data removal, transformations (e.g., centering or standardizing variables) or other changes prior to or following analysis  Report whether models meet assumptions for LMMs. Report if transformations were carried out in order to meet assumptions (e.g., log transformation of reaction time to meet the assumption that residuals are normally distributed). | 2.4.2. Quantitative Data Analysis  Supplementary Methods |
| Selection of fixed and random effects | Random effects are explicitly specified according to sampling units (e.g., patients, items), the data structure (e.g., repeated measures) and anticipated interactions between fixed effects and sampling units (e.g., intercepts only or intercepts and slopes). Fixed effects and covariates are specified from explicitly stated research questions and/or hypotheses.  Report the size of the sample analysed in terms of total number of data points and of sampling units (e.g., number of patients, number of items, number of other groups specified as random effects, such as classes of children). | 2.4.2. Quantitative Data Analysis  Supplementary Methods  Supplementary Table S4 |
| Model comparison | A clear statement of the methods by which models are compared/selected; e.g., simple to complex, covariates first, random effects first, fixed effects first etc.  Report comparison method (LRT, AIC, BIC) and justify the choice.  A complete report of all models compared (e.g., in appendices/supplementary data/analysis scripts) with model equations and the result of comparisons. An example table reporting model comparisons can be found in Appendix Table A5.1. | 2.4.2. Quantitative Data Analysis  Supplementary Methods  Supplementary Table S4 |
| Convergence issues | If models fail to converge, the approach taken to manage this should be comprehensively reported. This should include the formula for each model that did or did not converge and a rationale for a) the simplification method used and b) the final model reported. This may be most easily presented in an analysis script. | 2.4.2. Quantitative Data Analysis |
| Model* | Provide equation(s) that transparently define the reported model(s). An elegant way to do this is providing the model equation with the table that reports the model output | Tables 3-5 |
| Model output | Final model(s) reported in a table that includes all parameter estimates for fixed effects coefficients, standard errors and/or confidence intervals, associated test statistics and pvalues if used), random effects (standard deviation and/or variance for each random effect, correlations/covariances if modelled) and some measure of model fit (e.g. *R*-squared, correlation between fitted values and data). | Tables 3-5 (including all parameter estimates, test statistics, *p*-values, random effects, and model fit) |
| Data and code | Share coding script used to complete the analysis. Wherever possible share data that generated the reported results. | 2.4.2. Quantitative Data Analysis |

**Note.** AIC: Akaike Information criterion, BIC: Bayesion Information criterion, LMM: linear mixed model, LRT: likelihood-Ratio-Test.

**Supplementary Table S4**. Model building process.

| **Outcome** | **Predictor** | **Model name** | **Sampling units** | **Random effects** | **Model fit** |  |  | **LRT against needed** |  |  |
| --- | --- | --- | --- | --- | --- | --- | --- | --- | --- | --- |
|  |  |  |  |  | **AIC** | **BIC** | **LL** | ***df*** | **X²** | ***p*** |
| Self-reported success | Difference in baseline – regulation during feedback task | Model 1 | *N* total obs = 2183  *N* patients = 23  *N* sessions = 12  *N* trials = 12 | Intercept for participant, session, trial | Convergence warning – item variance close to zero |  |  |  |  |  |
|  |  | Model 2 |  | Intercept for participant, session | 6069.8 | 6098.2 | -3029.2 |  |  |  |
|  |  | Model 2_1 |  | Intercept for participant, trial | Convergence warning – item variance closed to zero. |  |  |  |  |  |
|  |  | Model 2_2 |  | Intercept for session, trial | Convergence warning – item variance close to zero |  |  |  |  |  |
|  |  | Model 3 |  | Intercept for participant | 6330.4 | 6353.2 | -3161.2 | 1 | 262.62 | <.001 |
| Self-reported success | Difference in baseline – regulation during transfer task | Model 1 | *N* total obs = 1086  *N* patients = 23  *N* sessions = 12  *N* trials = 12 | Intercept for participant, session, trial | Convergence warning – item variance close to zero |  |  |  |  |  |
|  |  | Model 2 |  | Intercept for participant, session | 2972.3 | 2997.3 | -3029.2 | -1481.2 | 105.35 | <.001 |
|  |  | Model 2_1 |  | Intercept for participant, trial | Convergence warning – item variance close to zero |  |  |  |  |  |
|  |  | Model 2_2 |  | Intercept for session, trial | Convergence warning – item variance close to zero |  |  |  |  |  |
|  |  | Model 3 |  | Intercept for participant | 3075.7 | 3095.6 | -3161.2 | -1533.8 | 262.62 | <.001 |
| Difference in baseline – regulation during feedback | Strategies | Model 1 | *N* total obs = 2454  *N* patients = 23  *N* sessions = 12  *N* trials = 12 | Intercept for participant, session, trial | Convergence warning – item variance close to zero |  |  |  |  |  |
|  |  | Model 2 |  | Intercept for participant, session | 6032.5 | 6096.4 | -3005.3 |  |  |  |
|  |  | Model 2_1 |  | Intercept for participant, trial | Convergence warning – item variance close to zero |  |  |  |  |  |
|  |  | Model 2_2 |  | Intercept for session, trial | Convergence warning – item variance close to zero |  |  |  |  |  |
|  |  | Model 3 |  | Intercept for participant | 6051.5 | 6104.2 | -3015.7 | 1 | 20.92 | <.001 |
| Difference in baseline – regulation during transfer | Strategies | Model 1 | *N* total obs = 1216  *N* patients = 23  *N* sessions = 12  *N* trials = 12 | Intercept for participant, session, trial | Convergence warning – item variance close to zero |  |  |  |  |  |
|  |  | Model 2 |  | Intercept for participant, session | 3012.5 | 3068.6 | -1495.2 |  |  |  |
|  |  | Model 2_1 |  | Intercept for participant, trial | Convergence warning – item variance close to zero |  |  |  |  |  |
|  |  | Model 2_2 |  | Intercept for session, trial | Convergence warning – item variance close to zero |  |  |  |  |  |
|  |  | Model 3 |  | Intercept for participant | 3028.3 | 3079.4 | -1504.2 | 1 | 17.82 | <.001 |
| Self-reported success | Strategies | Model 1 | *N* total obs = 186  *N* patients = 23 | Intercept for participant, session | 577.24 | 609.51 | -277.62 |  |  |  |
|  |  | Model 2 |  | Intercept for participant | 577.25 | 612.73 | 278.62 | 1 | 2.01 | .157 |

**Note.** AIC: Akaike Information criterion, BIC: Bayesion Information criterion, LL: LogLikelihood, LRT: likelihood-Ratio-Test. All models were specified with fixed effects, without any interaction, and with random intercepts.

.

# Supplementary References

Aho, K., Derryberry, D., & Peterson, T. (2014). Model selection for ecologists: the worldviews of AIC and BIC. *Ecology*, *95*(3), 631–636. https://doi.org/https://doi.org/10.1890/13-1452.1

American Psychiatric Association. (2013). *Diagnostic and statistical manual of mental disorders* (5th ed.). https://doi.org/10.1176/appi.books.9780890425596

Babiloni, F., Cincotti, F., Carducci, F., Rossini, P. M., & Babiloni, C. (2001). Spatial enhancement of EEG data by surface Laplacian estimation: The use of magnetic resonance imaging-based head models. *Clinical Neurophysiology*, *112*(5), 724–727. https://doi.org/10.1016/S1388-2457(01)00494-1

Barr, D. J., Levy, R., Scheepers, C., & Tily, H. J. (2013). Random effects structure for confirmatory hypothesis testing: Keep it maximal. *Journal of Memory and Language*, *68*(3), 255–278. https://doi.org/10.1016/j.jml.2012.11.001

Barton, K. (2020). *MuMIn: Multi-Model Inference* (R package version 1.43.17). https://cran.r-project.org/package=MuMIn

Bates, D., Mächler, M., Bolker, B. M., & Walker, S. C. (2015). Fitting linear mixed-effects models using lme4. *Journal of Statistical Software*, *67*(1). https://doi.org/10.18637/jss.v067.i01

Blechert, J., Meule, A., Busch, N. A., & Ohla, K. (2014). Food-pics: An image database for experimental research on eating and appetite. *Frontiers in Psychology*, *5*(617). https://doi.org/10.3389/fpsyg.2014.00617

Blume, M., Schmidt, R., & Hilbert, A. (2019). Abnormalities in the EEG power spectrum in bulimia nervosa, binge-eating disorder, and obesity: A systematic review. *European Eating Disorders Review*, *27*(2), 124–136. https://doi.org/10.1002/erv.2654

Blume, M., Schmidt, R., Schmidt, J., Martin, A., & Hilbert, A. (2022). EEG neurofeedback in the treatment of adults with binge-eating disorder: A randomized controlled pilot study. *Neurotherapeutics: The Journal of the American Society for Experimental NeuroTherapeutics*, *19*, 352–365. https://doi.org/10.1007/S13311-021-01149-9

Brauer, M., & Curtin, J. J. (2018). Linear mixed-effects models and the analysis of nonindependent data: A unified framework to analyze categorical and continuous independent variables that vary within-subjects and/or within-items. *Psychological Methods*, *23*(3), 389–411. https://doi.org/10.1037/met0000159

Chow, T., Javan, T., Ros, T., & Frewen, P. (2017). EEG dynamics of mindfulness meditation versus alpha neurofeedback: a sham-controlled study. *Mindfulness*, *8*(3), 572–584. https://doi.org/10.1007/s12671-016-0631-8

Drechsler, R., Straub, M., Doehnert, M., Heinrich, H., Steinhausen, H. C., & Brandeis, D. (2007). Controlled evaluation of a neurofeedback training of slow cortical potentials in children with Attention Deficit/Hyperactivity Disorder (ADHD). *Behavioral and Brain Functions*, *3*. https://doi.org/10.1186/1744-9081-3-35

Hilbert, A., Schmidt, R., Petroff, D., Prettin, C., Ehlis, A.-C., Lührs, M., & Rösch, S. (2023). Near-infrared spectroscopy neurofeedback for binge-eating disorder: an exploratory randomized comparison with electroencephalography neurofeedback*. Psychological Medicine*.

Hilbert, A., & Tuschen-Caffier, B. (2016). Eating disorder examination: Deutschsprachige Übersetzung [Eating disorder examination: German translation.] 2. Auflage. dgvt-Verlag. https://doi.org/10.1007/978-981-287-104-6_101

Hoedlmoser, K. (2007). *The significance of sleep-related theta synchronization for general memory abilities and the impact of neurofeedback on sleep [Unpublished doctoral dissertation]*. University of Salzburg.

Hoedlmoser, K., Pecherstorfer, T., Gruber, G., Anderer, P., Doppelmayr, M., Klimesch, W., & Schabus, M. (2008). Instrumental conditioning of human sensorimotor rhythm (12-15 Hz) and its impact on sleep as well as declarative learning. *Sleep*, *31*(10), 1401–1408. https://doi.org/10.5665/sleep/31.10.1401

Karhunen, L. J., Vanninen, E. J., Kuikka, J. T., Lappalainen, R. I., Tiihonen, J., & Uusitupa, M. I. J. (2000). Regional cerebral blood flow during exposure to food in obese binge eating women. *Psychiatry Research - Neuroimaging*, *99*(1), 29–42. https://doi.org/10.1016/S0925-4927(00)00053-6

Kober, S. E., Neuper, C., & Wood, G. (2020). Differential effects of up- and down-regulation of SMR Coherence on EEG Activity and memory performance: A neurofeedback training study. *Frontiers in Human Neuroscience*, *14*, 606684. https://doi.org/10.3389/fnhum.2020.606684

Kwon, M., Han, S., Kim, K., & Jun, S. C. (2019). Super-resolution for improving EEG spatial resolution using deep convolutional neural network—feasibility study. *Sensors (Basel, Switzerland)*, *19*(23). https://doi.org/10.3390/s19235317

Laird, N. M., & Ware, J. H. (1982). Random-effects models for longitudinal data. *Biometrics*, *38*(4), 963–974.

Meteyard, L., & Davies, R. (2020). Best practice guidance for linear mixed-effects models in psychological science. *Journal of Memory and Language*, *112*, 104092.

Nunez, P. L., Silberstein, R. B., Cadusch, P. J., Wijesinghe, R. S., Westdorp, A. F., & Srinivasan, R. (1994). A theoretical and experimental study of high resolution EEG based on surface Laplacians and cortical imaging. *Electroencephalography and Clinical Neurophysiology*, *90*(1), 40–57. https://doi.org/10.1016/0013-4694(94)90112-0

Tammela, L. I., Pääkkönen, A., Karhunen, L. J., Karhu, J., Uusitupa, M. I. J., & Kuikka, J. T. (2010). Brain electrical activity during food presentation in obese binge-eating women. *Clinical Physiology and Functional Imaging*, *30*(2), 135–140. https://doi.org/10.1111/j.1475-097X.2009.00916.x

Weber, L. A., Ethofer, T., & Ehlis, A. C. (2020). Predictors of neurofeedback training outcome: A systematic review. *NeuroImage: Clinical*, *27*, 102301. https://doi.org/10.1016/j.nicl.2020.102301
